# Acinar Cell-Derived Extracellular Vesicle MiRNA-183-5p Aggravates Acute Pancreatitis by Promoting M1 Macrophage Polarization Through Downregulation of FoxO1

**DOI:** 10.3389/fimmu.2022.869207

**Published:** 2022-07-13

**Authors:** De-sheng Tang, Feng Cao, Chang-sheng Yan, Ji-tao Cui, Xiao-yu Guo, Long Cheng, Le Li, Yi-long Li, Jia-min Ma, Kun Fang, Lei Gao, Nian-sheng Ren, Bei Sun, Gang Wang, Liang Ji

**Affiliations:** ^1^ Department of Pancreatic and Biliary Surgery, The First Affiliated Hospital of Harbin Medical University, Harbin, China; ^2^ Key Laboratory of Hepatosplenic Surgery, Ministry of Education, Harbin, China; ^3^ Department of General Surgery, Xuanwu Hospital, Capital Medical University, Beijing, China; ^4^ Clinical Center for Acute Pancreatitis, Capital Medical University, Beijing, China; ^5^ Department of Breast Surgery, The First Affiliated Hospital of Harbin Medical University, Harbin, China

**Keywords:** exosome, extracellular vesicles, inflammation, acute pancreatitis, macrophage polarization

## Abstract

Acute pancreatitis (AP) is a common cause of a clinically acute abdomen. Crosstalk between acinar cells and leukocytes (especially macrophages) plays an important role in the development of AP. However, the mechanism mediating the interaction between acinar cells and macrophages is still unclear. This study was performed to explore the role of acinar cell extracellular vesicles (EVs) in the crosstalk between acinar cells and macrophages involved in the pathogenesis of AP. EVs derived from caerulein-treated acinar cells induced macrophage infiltration and aggravated pancreatitis in an AP rat model. Further research showed that acinar cell-derived EV miR-183-5p led to M1 macrophage polarization by downregulating forkhead box protein O1 (FoxO1), and a dual-luciferase reporter assay confirmed that FoxO1 was directly inhibited by miR-183-5p. In addition, acinar cell-derived EV miR-183-5p reduced macrophage phagocytosis. Acinar cell-derived EV miR-183-5p promoted the pancreatic infiltration of M1 macrophages and increased local and systemic damage *in vivo*. Subsequently, miR-183-5p overexpression in macrophages induced acinar cell damage and trypsin activation, thus further exacerbating the disease. In clinical samples, elevated miR-183-5p levels were detected in serum EVs and positively correlated with the severity of AP. EV miR-183-5p might play an important role in the development of AP by facilitating M1 macrophage polarization, providing a new insight into the diagnosis and targeted management of pancreatitis.

Graphical abstract of the present study. In our caerulein-induced AP model, miR-183-5p was upregulated in injured acinar cells and transported by EVs to macrophages. miR-183-5p could induce M1 macrophage polarization through downregulation of FoxO1 and the release of inflammatory cytokines, which could aggravate AP-related injuries. Therefore, a vicious cycle might exist between injured ACs and M1 macrophage polarization, which is fulfilled by EV-transported miR-183-5p, leading to sustainable and progressive AP-related injuries.

## Introduction

Acute pancreatitis (AP) commonly presents with an acute abdomen, with or without local or/and systemic disorders. Although mild AP may be resolved with a good prognosis, approximately 1/5 of the patients will develop a severe subtype with a mortality rate of greater than 20%. Therefore, severe AP seriously threatens the health of patients, and inhibiting the aggravation of pancreatitis is a key strategy. Researchers have postulated that abnormal trypsin activation is the initial event in the pathogenesis of AP (1). However, AP is characterized by sustained local and systemic inflammatory injuries, which involve a number of organs and systems. The connection between the initial acinar cell damage and the sustainable AP-related damage and the underlying mechanisms are still largely unknown.

Damage-associated molecular patterns (DAMPs) that are released from acinar cells beginning at the onset of AP aggravate pancreatitis by recruiting immune cells and activating inflammatory responses. Macrophages, especially M1 macrophages, are rapidly activated in early-stage AP and aggravate pancreatitis by releasing large amounts of inflammatory factors and interleukins (2). Acinar cell damage and M1 macrophage polarization trigger intense local inflammation and subsequently lead to the development of systemic inflammatory response syndrome (SIRS) and multiple organ dysfunction syndrome (MODS) by interacting with each other to form a positive feedback loop (3–5). However, during the development of AP, the mechanism by which acinar cells regulate macrophages is not fully understood.

Extracellular vesicles (EVs) are produced by cells and carry proteins and nucleic acids to regulate the host immune response by mediating the transfer of substances between cells; thus, EVs play an important role in the occurrence and development of inflammatory diseases (6–8). Previous studies have shown that a variety of microRNAs (miRNAs) are abnormally expressed in AP, which may aggravate pancreatitis by regulating the apoptosis of acinar cells, the abnormal activation of trypsin and the proportion of immune cells (9–11). EVs derived from Klotho-overexpressing mesenchymal stem cells reverse acinar cell apoptosis and reduce pancreatitis (12). As a significant component within EVs, miRNAs have been extensively studied to explore their functions in the development of AP. Notably, miRNAs derived from EVs may be involved in regulating the occurrence and development of AP. The crosstalk between acinar cells and macrophages affects the damage and repair of AP (5). However, the regulation of EVs and the molecular mechanism by which they are involved in the crosstalk between acinar cells and macrophages at an early stage of AP have not been reported in the literature. In this study, we hypothesized that EV miRNAs might act as a bridge that mediates the crosstalk between acinar cells and macrophages in the process of AP.

## Materials and Methods

### Ethics Statement and Preparation of Human Blood Samples

All human blood samples were obtained as discarded clinical samples at the First Clinical College of Harbin Medical University. Procedures involving human blood samples were conducted in accordance with the Declaration of Helsinki and approved by the Ethics Committee of the First Affiliated Hospital of Harbin Medical University (Protocol Number: 2021IIT060). All subjects signed an informed consent form.

### Animal Models of AP

Thirty-two male Wistar rats (180–200 grams) were provided by the Animal Research Center of the First Affiliated Hospital of Harbin Medical University (Harbin, China). The animal care and experimental protocols were all approved by the Institutional Animal Care and Use Committee of Harbin Medical University. All animals were fasted for 8 h before modeling and were maintained under a 12-h light/dark cycle. A rat model of AP was established. First, the rats were anesthetized by administering a intraperitoneal injection of 10 mg/kg sodium pentobarbital (Sigma–Aldrich, MO, USA) dissolved in 300 µl of saline. Then, the abdomen was opened along the midline, and the distal pancreaticobiliary duct was ligated. The rat AP model was established through the retrograde injection of 300 μl of 3.5% sodium taurocholate (Sigma-Aldrich) into the pancreatic duct. Two hours after the rat AP model was established, the rats were allocated into the following four groups to explore the effect of EVs on macrophages *in vivo*: sham (sham operation), AP (AP model), AP+Ctrl-ev and AP+Cae-ev (n=4 rats per group). Rats in the AP+Ctrl-ev group received EVs from AR42J cells (ACs) treated with phosphate-buffered saline (PBS) and rats in the AP+Cae-ev groups received EVs from ACs treated caerulein. Afterward, 200 μg (4x10^11^ particles) of EVs were intraperitoneally injected. Six hours later, we sacrificed the rats and harvested the tissue. We allocated rats into another four groups 2 hours after the rat AP model was established to confirm the role of miR-183-5p in promoting M1 macrophage polarization *in vivo*: NC-mimic, NC-inhibitor, miR-183-5p mimic and miR-183-5p inhibitor (n = 4 rats per group). The AP rats in the miR-183-5p mimic or inhibitor groups were treated with EVs from ACs that were pre-transfected with the miR-183-5p mimic or inhibitor, respectively. The AP rats were sacrificed at 6 hours after the EVs (4x10^11^ particles) were intraperitoneally injected.

### Cell Culture

NR8383 macrophages (Shanghai Chinese Academy of Sciences Cell Bank, Shanghai, China) were cultured in DMEM-F12 (Thermo Fisher Scientific, MA, USA) supplemented with 20% fetal bovine serum (FBS, Gibco, USA) and a 1% penicillin–streptomycin solution (Procell, Wuhan, China). AR42J cells (ACs, Shanghai Chinese Academy of Sciences Cell Bank) were cultured in DMEM supplemented with a 1% penicillin–streptomycin solution and 10% FBS. Bone marrow-derived macrophages (BMDMs) were obtained from the femurs of wild-type Wistar rats. After 10 days of culturing in DMEM supplemented with a 1% penicillin–streptomycin solution, 10% FBS and 10 ng/ml recombinant rat macrophage colony-stimulating factor (M-CSF; PeproTech, NJ, USA), BMDMs were artificially differentiated into macrophages.

### Cocultivation Experiment

The ACs were first treated with PBS or caerulein (1x10^-8^ mol/L, Sigma–Aldrich) or GW4869 (1×10^-5^ mol/L, Sigma–Aldrich) combined with caerulein for 6 hours before co-culture with macrophages to study whether caerulein affected miR-183-5p loading into EVs and transfer to recipient macrophages. Specifically, ACs were seeded in the upper chamber of 6-well Transwell permeable support coculture systems with a 0.4 µm pore filter (Corning, New York, USA). NR8383 macrophages were seeded into the lower chamber of a 6-well plate and co-cultured with ACs in the upper chamber for 12 h, and DMEM/F12 was used as the culture medium. The expression of miR-183-5p in ACs and macrophages was measured. We cocultured these NR8383 macrophages with caerulein-treated ACs for 12 h to determine whether macrophages overexpressing miR-183-5p damaged ACs. The NR8383 macrophages overexpressing miR-183-5p were plated in the upper chamber of the coculture system, whereas the ACs were plated in the lower chambers of the 6-well plate, and DMEM/F12 was used as the culture medium.

### Cell Transfection

The NR8383 cells and BMDMs were plated in a 6-well plate. When cells reached 50% confluence, they were transfected with 10 nM NC-mimic, miR-183-5p mimic, NC-inhibitor, miR-183-5p inhibitor, siFoxO1 or si-NC (RiboBio, Guangzhou, China). Lipofectamine 3000 (11668019, Thermo) was used for transfection experiments according to the product instructions.

### EV Extraction

Forty-eight hours before EV extraction from the cell culture supernatant, the FBS in the AC culture medium was replaced with EV-depleted FBS (System Biosciences, California, USA). Caerulein, a decapeptide molecule extracted from the skin of the *Hyla caerulea* frog, is a cholecystokinin analog that acts on pancreatic acinar cells and causes the secretion of a large amount of digestive enzymes and pancreatic juice, resulting in AP. After ACs were treated with PBS or caerulein (1x10^-8^ mol/L) for 6 hours, the medium was refreshed, and the cells were cultured for another 48 h. EVs were purified by differential ultracentrifugation. In short, the samples were centrifuged at 300×g and 2000×g for 20 min each and then at 10,000×g for 30 min, and the collected supernatant was centrifuged at 100,000×g for 70 min. The precipitates were washed with PBS, centrifuged at 100,000 × *g* for 70 min and resuspended in 100 μl of PBS. The process of EV extraction from ACs with miR-183-5p overexpression and knockdown is described below. After the transfection of miR-183-5p mimic and inhibitor into ACs using the aforementioned transfection method, the culture was continued for 48 hours, and EVs were extracted from the cell supernatant by ultracentrifugation. Three milliliters of venous blood were collected from 15 pairs of patients with AP and healthy controls to extract EVs from sera. Serum was separated by centrifugation at 3,000 rpm for 10 min at 4°C to remove blood cells and debris. Finally, a 1 ml serum supernatant sample was diluted with 5 ml of PBS, and EVs were purified by differential ultracentrifugation.

### Transmission Electron Microscopy (TEM)

All EV samples subjected to TEM were diluted 10-fold with PBS and then passed through 200-mesh nickel grids to remove any potential residual organelle debris. After being stained with 4% phosphotungstic acid for 3 min at room temperature, the samples were placed on filter paper and air-dried. EV morphology and size were imaged with a transmission electron microscope (HT7700, Hitachi, Japan).

### Nanoparticle Tracking Analysis (NTA)

NTA was performed with a nanoparticle size potentiometer (Zetasizer Ultra, United Kingdom). Briefly, 1 ml of sterile PBS-diluted EVs was dropped into the machine, and the diffusion rate of the particles in the solution was converted to the particle size of EVs.

### EV Labeling and Tracking

ACs were stained with PKH67 (MIDI67, Sigma–Aldrich) in the dark at room temperature, and the unbound dye was removed by washing with PBS. EVs were separated by ultracentrifugation using the abovementioned method and added to BMDMs. After 24 h of culture, EV uptake by BMDMs was observed under a microscope. AP rats were sacrificed 6 h after treatment with PKH67-labeled EVs, and the distribution of the EVs in various organs was observed using frozen sections.

### EV miRNA Expression Profiling

EVs were isolated from ACs treated with or without caerulein, and miRNAs were sequenced using a second-generation high-throughput sequencer (Illumina, CA, USA). The final data were compared with the reference genome using the BWA software to obtain genomic expression profiles.

### Luciferase Reporter Assay

To determine whether FoxO1 is a direct target of miR-183-5p, luciferase reporter constructs (3′UTR-NC, 3′UTR-FoxO1, and 3′UTR-FoxO1-mutant), miRNA (miRNA-NC or miR-183-5p) and Renilla luciferase (GeneChem, Shanghai, China) were transfected into 293T cells using Lipofectamine 3000 (Thermo Fisher Scientific). Cells were harvested at 48 h after transfection, and luciferase activity was determined using a dual-luciferase assay system (Promega, WI, USA) following a standard procedure.

### RNA Isolation and Extraction

Total RNA was isolated using an RNA extraction kit (Axygen, CA, USA). MiRNA and mRNA were transcribed into cDNA by a reverse transcription kit (Toyobo, Japan). The cDNA template was analyzed by qRT-PCR using SYBR Green assays (Roche, Germany). GAPDH was used as an internal reference for mRNA, and U6 was used as an internal reference for miRNA. PCR was performed using FastStart Universal SYBR Green Master Mix and a 7500 Real-Time PCR System (Basel, Switzerland). The absolute quantification of miR-183-5p in samples is described below. Briefly, a standard curve of the logarithmic values of miR-183-5p concentrations and the CT value was prepared by stepwise dilution of the miR-183-5p standard RNA (RiboBio, Guangzhou, China). By measuring the CT value of the sample, the absolute level of miR-183-5p in the sample was calculated. The primer sequences are shown in **Supplemental Table 1**.

### Western Blot Analysis

Samples were lysed in RIPA lysis buffer supplemented with protease and phosphatase inhibitors, and the protein concentration was measured by the BCA assay (Beyotime, Shanghai, China). Protein samples were separated on 10% SDS-PAGE gels, and the separated proteins were transferred onto nitrocellulose membranes, which were blocked with 5% milk and then incubated overnight with the following antibodies: anti-Alix (1:1,000, ab275377, Abcam, UK), anti-TSG101 (1:1,000, ab125011, Abcam), anti-CD63 (1:1,000, A5271, ABclonal), anti-FoxO1 (1:1,000, C29H4, Cell Signaling Technology), anti-P-FoxO1 (1:1,000, 9461, Cell Signaling Technology), β-actin (1:2,000, BA2305, Boster), anti-iNOS (1:1,000, BA0362, Boster), anti-Arg-1 (1:1,000, 93668, Cell Signaling Technology), anti-P65 (1:2,000, 8242S, Cell Signaling Technology), anti-P-P65 (1:1,000, 93H1, Cell Signaling Technology) and anti-TNF-α (1:1,000, ab205587, Abcam). After incubation for 1 h with the corresponding fluorescent secondary antibody.

### Hematoxylin-eosin (HE) Staining

After pancreatic tissue samples were embedded using a cryostat, the tissue was sliced at 10 μm thickness and placed on a glass slide. HE-stained paraffin sections were subjected to xylene dewaxing, hydration in an ethanol gradient, HE staining, dehydration in an alcohol gradient and xylene, and mounting. Tissue damage was observed under the microscope. We applied the scoring system defined by Kusshe et al. (13), and the final score for each group was calculated.

### Immunofluorescence (IF)

The IF protocol has been previously described (14). Briefly, the pancreatic tissues were blocked with 5% donkey serum containing 0.1% Triton X-100 for 1 h at room temperature and then incubated overnight at 4°C with the following primary antibodies: CD86 (1:200, BU63, Novus, CO, USA), iNOS (1:100, GB11119, Servicebio) and CD68 (1:200, KP1, Novus). Subsequently, the slides were washed with PBS (0.01 M, pH 7.4) and incubated with the corresponding fluorophore-conjugated secondary antibodies, namely, goat anti-rabbit IgG (1:100, A27039, Invitrogen) and goat anti-mouse IgG (1:100, ab6785, Abcam), at 37°C for 1 h. 4′,6-Diamidino-2-phenylindole (DAPI, Abcam) was used for nuclear staining. When cells were stained, BMDMs overexpressing and inhibiting miR-183-5p were blocked with 5% serum and permeabilized with 0.3% Triton X-100, then incubated with the FoxO1 antibody (1:100, C29H4, Cell Signaling Technology) overnight at 4°C. Finally, BMDMs were incubated with appropriate secondary antibodies and DAPI. After staining, BMDMs were examined using a fluorescence microscope.

### Serum and Tissue Assays

Amylase, lipase, creatinine and urea nitrogen levels in the blood samples and myeloperoxidase levels in lung tissue were measured using standard diagnostic kits (Jiancheng Biotech, Nanjing, China). Tumor necrosis factor-α (TNF-α) and interleukin-6 (IL-6) levels in the pancreatic tissue samples and peripheral blood were measured using standard diagnostic kits (R&D Systems, Minneapolis, MN, USA) according to the manufacturer’s instructions.

### Flow Cytometry

Single-cell suspensions of NR8383 cells and BMDMs were prepared as previously described. BMDMs were incubated first with CD16/CD32 (Thermo Fisher Scientific) and then with FITC-CD68 (MA5-28262, Thermo Fisher Scientific) and PERCPEF710-CD11b/c (46-0110-82, Thermo Fisher Scientific). The percentage of double-positive cells indicates the purity of macrophages. To identify macrophage polarization, cells were first incubated with CD16/CD32, followed by PE-CD86 (12-0860-83, Thermo Fisher Scientific) and CD163 (PA5-78961, Thermo Fisher Scientific). Finally, the cells were incubated in the dark for 1 h with APC (A-10931, Thermo Fisher Scientific). Apoptosis was determined by an Annexin V-FITC/PI double staining cell apoptosis detection kit (Sevenbio, Beijing, China) following the manufacturer’s instructions. Each sample was analyzed by flow cytometry and FlowJo v.10 software.

### Phagocytic Assay

Macrophages were seeded into a 6-well plate (20 × 10^5^ cells per well). Fluorescein isothiocyanate (FITC)-OVA (100 μg/ml, Solarbio, Beijing, China) was added to the 6-well plate, which was incubated for 6 h at 37°C in the dark and washed 3 times with 0.01 M PBS, pH 7.2. Then, the fluorescence intensity of each sample was measured on a flow cytometer.

### Statistical Analysis

All data were processed using GraphPad Prism 7.0. Data are presented as the mean ± standard deviation. Paired data in two groups were compared using a *t*-test. One-way ANOVA followed by the Bonferroni correction was performed to compare count data in more than two groups. A *P*-value of <0.05 was considered to be statistically significant.

## Results

### EVs from Caerulein-Treated ACs Aggravate AP

**Figure 1:**
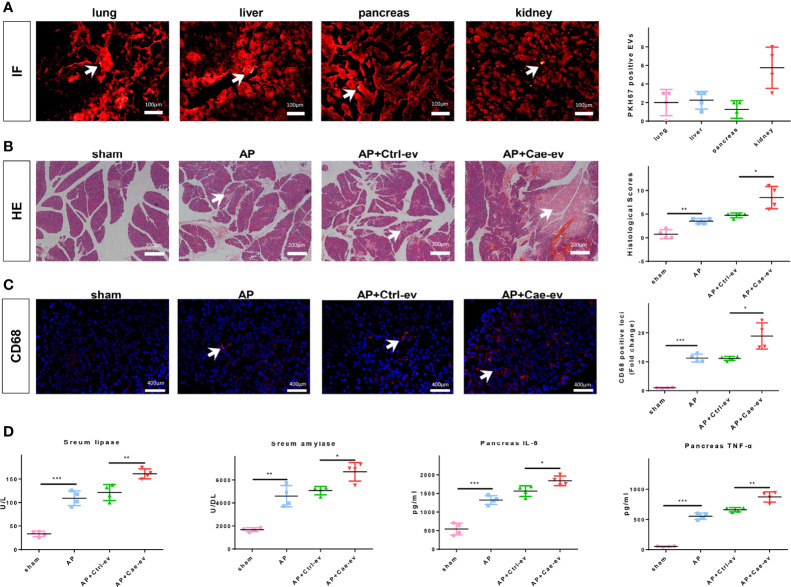
EVs from caerulein-treated ACs aggravate AP. **(A)** Photographs of IF-stained sections of AP rat lung, liver, pancreas, and kidney samples harvested 6 h after EV injection. The number of PKH67-labeled EVs in the same field of view from the four groups was determined. EVs are labeled with white arrows. Scale bar, 100 μm. **(B)** Representative photographs and histological scores of HE-stained pancreatic sections harvested from the rats in the 4 groups at 6 h after EV injection: sham, AP, AP+Ctrl-ev and AP+Cae-ev groups. Pancreatic injury is indicated by the white arrows. Scale bar, 200 μm. **(C)** Microscopy images of the distribution of CD68 immunostaining in pancreatic sections from the aforementioned groups of AP rats. Scale bars, 200 μm. The fold change in the number of CD68 positive macrophages in the four groups was determined. Scale bar, 400 μm. **(D)** Amylase and lipase levels in the blood and inflammatory cytokine levels in the pancreas were determined by ELISA. Data are presented as the mean ± SD. All experiments were repeated three times. *p < 0.05, **p < 0.01, ***p < 0.001. *AP:* AP rats, *AP+Ctrl-ev:* AP rats treated with EVs from PBS-treated acinar cells, *AP+Cae-ev:* AP rats treated with EVs from caerulein-treated acinar cells.

We intraperitoneally injected PKH67-labeled EVs from ACs into AP rats to evaluate whether EVs reached the pancreatic tissue and aggravated pancreatitis. IF staining showed that PKH67-labeled EVs reached the pancreatic tissue, as well as the lungs, liver, and kidneys, which are vulnerable to the attack of pancreatitis (**Figure 1A**). Subsequently, we extracted EVs from ACs treated with PBS or caerulein and injected them into AP rats to confirm the effect of AC-derived EVs on pancreatitis. Caerulein-treated AC-derived EVs did not increase MPO (neutrophil marker) levels in the pancreas (**Supplemental Figure 1A**). Interestingly, EVs from caerulein-treated ACs aggravated pancreatic tissue damage (**Figure 1B**) and increased CD68-positive macrophages infiltration (**Figure 1C**). Subsequently, we measured the levels of lipase and amylase in the blood and inflammatory cytokines in the pancreas, and the results showed that caerulein-treated AC-derived EVs increased lipase, amylase, IL-6 and TNF-α levels (**Figure 1D**). Additionally, caerulein-treated AC-derived EVs also increased the levels of IL-6 and TNF-α in peripheral blood (**Supplemental Figure 1B**). Based on these results, EVs from caerulein-treated ACs aggravate AP.

### EVs Derived from Caerulein-Treated ACs Promote M1 Macrophage Polarization

**Figure 2:**
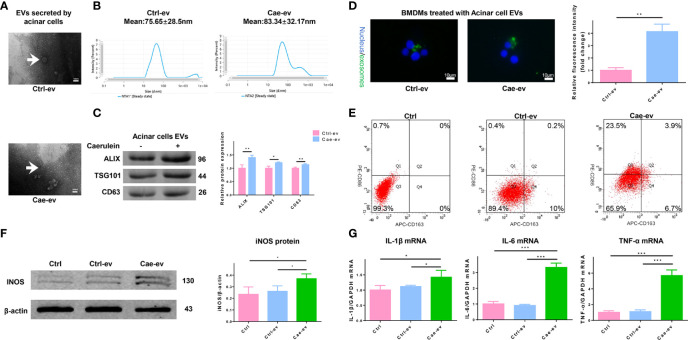
EVs from caerulein-treated ACs promote M1 macrophage polarization. **(A)** Representative electron micrograph of EVs purified from AC supernatant from the Ctrl-ev and Cae-ev groups. EVs are indicated by the white arrows. Scale bar, 100 nm. **(B)** NTA-based determination of the diameter of EVs in the Ctrl-ev and Cae-ev groups. **(C)** Protein expression and analysis of EV markers (including Alix, TSG101 and CD63) in EVs from ACs treated with or without caerulein by western blotting. **(D)** Macrophages were treated for 12 h with PKH67-labeled EVs from the Ctrl-ev and Cae-ev groups. The relative fluorescence intensity was calculated. Scale bar, 10 μm. **(E)** CD86 (M1 macrophage marker) and CD163 (M2 macrophage marker) expression levels in macrophages treated with Ctrl-ev and Cae-ev were detected by flow cytometry. **(F)** Protein expression and analysis of iNOS in macrophages treated with EVs from PBS-treated ACs and caerulein-treated ACs using western blotting. **(G)** Inflammatory cytokine mRNA expression (IL-1β, IL-6 and TNF-α) in macrophages was detected by qRT-PCR. Data are presented as the mean ± SD. All experiments were repeated three times. *p < 0.05, **p < 0.01, ***p < 0.001. *NTA:* nanoparticle tracking analysis, *Ctrl-ev:* EVs derived from PBS-treated acinar cells, *Cae-ev:* EVs derived from caerulein-treated acinar cells.

We next evaluated the characteristics of EVs isolated and purified from ACs. First, the size and morphology of EVs secreted from ACs treated with PBS or caerulein were determined by TEM, and the results revealed discoid vesicles between 50 and 150 nm in diameter (**Figure 2A**). NTA showed that the mean particle size of EVs purified from ACs treated with or without caerulein was 83.34 ± 32.17 nm and 75.65 ± 28.5 nm, respectively (**Figure 2B**). The EV protein markers Alix, TSG101 and CD63 were also used for western blotting to identify EVs (**Figure 2C**). The extracted material was EVs. Subsequently, we incubated PKH67-stained EVs with BMDMs to ascertain whether EVs were internalized by macrophages. The fluorescence analysis showed that AC-derived EVs were internalized by BMDMs, and this phenomenon was more obvious for EVs derived from caerulein-treated ACs (**Figure 2D**). Next, we investigated whether AC-derived EVs impact macrophages by extracting monocytes from the rat bone marrow and transforming them into macrophages by treatment with M-CSF. Flow cytometry showed that 78.8% of the macrophage population was positive for both the macrophage markers CD68 and CD11B/C (**Supplemental Figure 2A**). After treating macrophages with PBS or caerulein-treated AC-derived EVs for 12 h, the proportion of macrophages treated with EVs from caerulein-treated ACs that were positive for CD86 (an M1 macrophage marker) was significantly increased (**Figure 2E**). Western blot analysis showed a significant increase in the expression of inducible nitric oxide synthase (iNOS) (an M1 macrophage marker) in BMDMs treated with EVs from caerulein-treated ACs (**Figure 2F**). qRT-PCR showed the significant upregulation of the expression of inflammatory cytokines, including IL-1β, IL-6 and TNF-α, in BMDMs treated with EVs from caerulein-treated ACs (**Figure 2G**). Thus, EVs derived from caerulein-treated ACs promote M1 macrophage polarization.

### High miR-183-5p Expression is Detected in EVs Derived from Caerulein-Treated ACs

**Figure 3:**
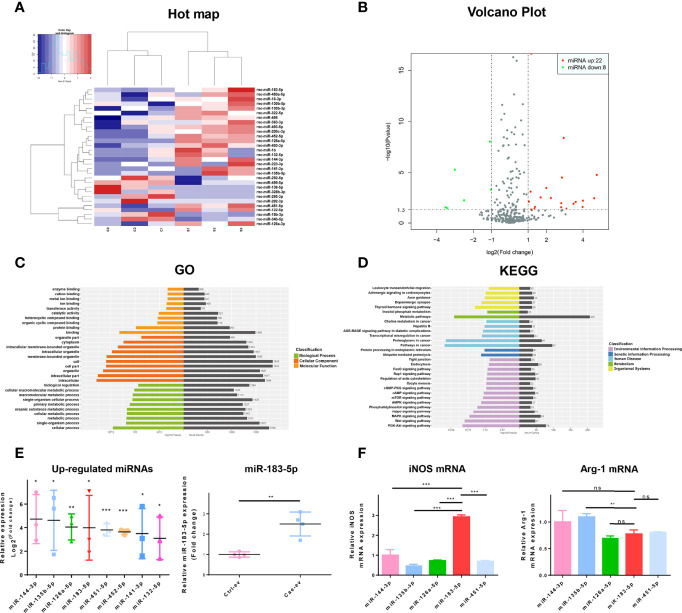
MiR-183-5p is highly expressed in caerulein-induced ACs. **(A)** Heat map of differentially expressed EV miRNAs. The expression cluster shows the expression level of EV miRNAs on the left of the figure. Red indicates high expression, and blue represents low expression. C1, C2 and C3 represent EVs secreted by saline-treated ACs; S1, S2, and S3 represent EVs secreted by caerulein-treated ACs. **(B)** The volcano plot of miRNAs from AC-derived EVs. Green dots represent downregulated miRNAs, and red dots represent upregulated miRNAs. **(C)** GO functional enrichment analysis of target genes of upregulated miRNAs. The vertical axis shows the GO term, and the horizontal axis shows the P*-*value and gene number. **(D)** KEGG pathway analysis revealed the top 30 enriched pathways involving the target genes of upregulated miRNAs. **(E)** The expression of the top eight upregulated miRNAs in EVs measured by sequencing. The expression of miR-183-5p in Ctrl-ev and Cae-ev was detected using qRT-PCR **(F)** After the overexpression of miRNAs in BMDMs, iNOS and Arg-1 mRNA expression in BMDMs were detected by qRT-PCR. Data are presented as the mean ± SD. All experiments were repeated three times. *p < 0.05, **p < 0.01, ***p < 0.001. *KEGG* Kyoto Encyclopedia of Genes and Genomes, *GO* Gene Ontology. *Ctrl-ev:* EVs derived from PBS-treated acinar cells, *Cae-ev:* EVs derived from caerulein-treated acinar cells.

We analyzed the differentially expressed miRNAs in EVs from ACs treated with PBS or caerulein by high-throughput sequencing to explore whether EV miRNAs derived from AC affected AP. Compared with EVs from ACs treated with PBS, those from caerulein-treated ACs contained 30 (22 upregulated and 8 downregulated) differentially expressed miRNAs (**Figures 3A, B**). Kyoto Encyclopedia of Genes and Genomes (KEGG) pathway annotation and Gene Ontology (GO) analyses were performed on the upregulated miRNAs to identify the functions of these differentially expressed miRNAs in EVs. GO analysis showed that the upregulated miRNAs mainly regulate the binding of intracellular proteins (**Figure 3C**). The KEGG annotation analysis showed that the upregulated miRNAs may play a role in inflammation by regulating leukocytes. In addition, this analysis predicted the potential involvement of the FoxO signaling pathways (**Figure 3D**). Sequencing results showed that the top 8 significantly upregulated miRNAs were detected in EVs from caerulein-treated ACs. The expression of miR-183-5p in EVs derived from ACs treated with PBS (Ctrl-ev) and caerulein (Cae-ev) was verified by qRT-PCR, and miR-183-5p was upregulated in Cae-ev, which again suggested that the sequencing was accurate (**Figure 3E**). Subsequently, we overexpressed the top 5 differentially expressed miRNAs in macrophages (**Supplemental Figure 3A**), and iNOS and arginase 1 (Arg-1) expression levels were verified by qRT-PCR. The results showed a significant increase in iNOS expression in macrophages overexpressing miR-183-5p (**Figure 3F**). Thus, miR-183-5p may induce M1 macrophage polarization.

### AC-Derived EV miR-183-5p Promotes M1 Macrophage Polarization

**Figure 4:**
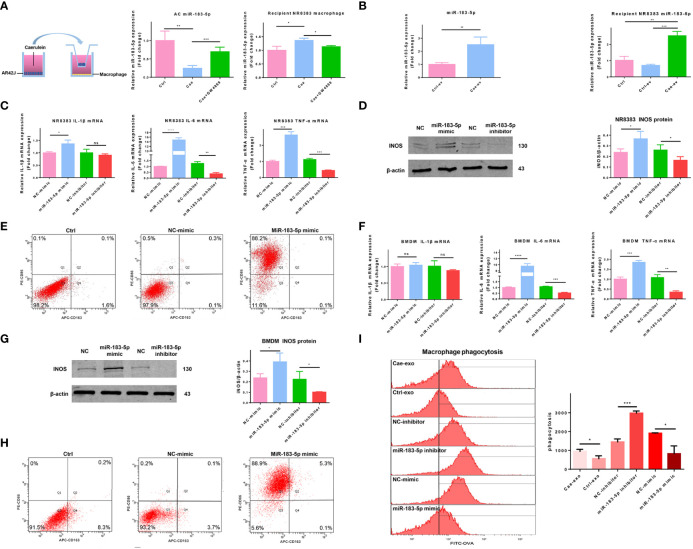
AC-derived EV miR-183-5p promotes M1 macrophage polarization. **(A)** Expression of miR-183-5p in ACs and NR8383 macrophages from the Ctrl, Cae, or Cae+GW4869 group in a co-culture system. **(B)** Expression of miR-183-5p in NR8383 macrophages treated with Ctrl-ev or Cae-ev. **(C)** Levels of inflammatory cytokines (IL-1β, IL-6 and TNF-α) in NR8383 macrophages transfected with NC-mimic, miR-183-5p mimic, NC-inhibitor and miR-183-5p inhibitor. **(D)** Protein expression and analysis of iNOS in the aforementioned groups of NR8383 macrophages by western blotting. **(E)** The expression of CD86 and CD163 in the aforementioned groups of NR8383 cells was detected by flow cytometry. **(F)** Levels of inflammatory cytokines (IL-1β, IL-6 and TNF-α) in BMDMs transfected with NC-mimic, miR-183-5p mimic, NC-inhibitor and miR-183-5p inhibitor. **(G)** Protein expression and analysis of iNOS in the aforementioned groups of BMDMs by western blotting. **(H)** The expression of CD86 and CD163 in BMDMs transfected with NC-mimic and miR-183-5p mimic was detected using flow cytometry. **(I)** The phagocytic activity of macrophages in the Cae-ev, Ctrl-ev, miR-183-5p mimic and miR-183-5p inhibitor groups was quantified by flow cytometry. Data are presented as the mean ± SD. All experiments were repeated three times. *p < 0.05, **p < 0.01, ***p < 0.001. *Ctrl:* PBS-treated acinar cells, *Cae:* caerulein-treated acinar cells, *Cae+GW4869:* EVs from acinar cells co-treated with caerulein and GW4869, *Ctrl-ev:* EVs derived from PBS-treated acinar cells, *Cae-ev:* EVs derived from caerulein-treated acinar cells, *NC-mimic:* macrophages transfected with NC-mimic, *miR-183-5p mimic:* macrophages transfected with miR-183-5p mimic, *NC-inhibitor:* macrophages transfected with NC-inhibitor, *miR-183-5p inhibitor:* macrophages transfected with miR-183-5p inhibitor, and *BMDMs:* bone marrow-derived macrophages.

We chose the well-known EV inhibitor GW4869 to inhibit the secretion of EVs and better determine the function of EVs. The inhibitory effect of GW4869 on EVs secreted by ACs was confirmed by western blotting. The amount of EVs released by GW4869-treated ACs was significantly reduced, and the levels of TSG101 and Alix in lysates of EVs released by GW4869-treated ACs were also reduced (**Supplemental Figure 4A**). We explored whether EV miR-183-5p from ACs reached macrophages and promoted M1 macrophage polarization by treating ACs with PBS, caerulein, or GW4869 combined with caerulein for 6 hours and co-cultured them with NR8383 macrophages. Cells in the two chambers were separated by a 0.4 μm Transwell filter. The caerulein-treated ACs released more miR-183-5p and the release of miR-183-5p was significantly inhibited by GW4869. Therefore, ACs stimulated with caerulein secrete miR-183-5p-rich EVs that are taken up by macrophages, and EVs play a major role in the process of transporting miR-183-5p (**Figure 4A**). We added the extracted EVs to NR8383 macrophages and incubated them for 12 hours to further assess the effect of EVs on macrophages. qRT-PCR showed higher miR-183-5p expression in Cae-ev than in Ctrl-ev, and its expression was significantly increased in Cae-ev-treated NR8383 macrophages, further confirming that EVs play a key role in transporting miR-183-5p to macrophages (**Figure 4B**). We transfected an miR-183-5p mimic and miR-183-5p inhibitor into NR8383 macrophages and BMDMs to investigate whether AC-derived EV miR-183-5p mediated M1 macrophage polarization. The mRNA expression levels of IL-6 and TNF-α increased significantly in NR8383 macrophages overexpressing miR-183-5p (mimic group) but decreased in the miR-183-5p inhibitor group (**Figure 4C**). Western blot analysis showed that iNOS expression was significantly upregulated in NR8383 macrophages overexpressing miR-183-5p and suppressed in the miR-183-5p inhibitor group (**Figure 4D**). The expression of Arg-1 was not significantly changed in NR8383 macrophages with miR-183-5p overexpression and knockdown (**Supplemental Figure 4B**). Flow cytometry showed that the overexpression of miR-183-5p in NR8383 cells significantly increased the proportion of CD86-positive cells (**Figure 4E**). Similar results were confirmed in isolated BMDMs (**Figures 4F–H**). In addition, OVA-FITC staining showed that miR-183-5p overexpression reduced macrophage-mediated phagocytosis (**Figure 4I**). Therefore, miR-183-5p plays a role in promoting M1 polarization and reducing macrophage phagocytosis.

### AC-Derived EV MiR-183-5p Promotes M1 Macrophage Polarization by Targeting FoxO1

**Figure 5:**
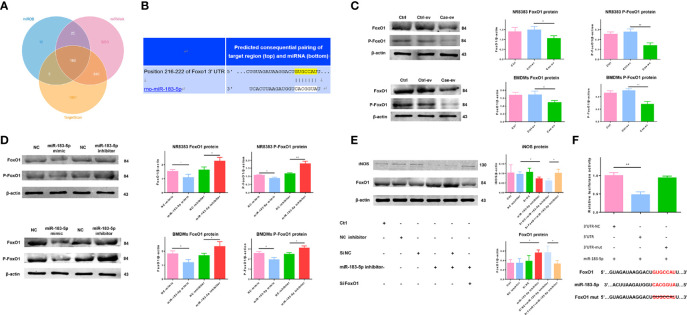
AC-derived EV miR-183-5p induces macrophage M1 polarization by targeting FoxO1. **(A)** Venn diagram showing the number of potential common target genes identified by three bioinformatics analysis tools. **(B)** The presence of a complementary miR-183-5p sequence in the 3′-UTR of FoxO1 mRNA was predicted by TargetScan. **(C-D)** Western blotting: **(C)** levels and analysis of the FoxO1 and P-FoxO1 proteins in NR8383 macrophages and BMDMs treated with Ctrl-ev and Cae-ev; **(D)** levels and analysis of the FoxO1 and P-FoxO1 proteins in NR8383 macrophages and BMDMs transfected with NC-mimic, miR-183-5p mimic, NC-inhibitor and miR-183-5p inhibitor. **(E)** Levels and analysis of the FoxO1 and iNOS proteins in BMDMs transfected with the NC inhibitor, miR-183-5p inhibitor, si-NC+miR-183-5p inhibitor and siFoxO1+miR-183-5p inhibitor. **(F)** A luciferase reporter assay was performed with 3′UTR-NC (negative control), 3′UTR-FoxO1 and 3′UTR-FoxO1-mutant. The miR-183-5p overexpression plasmid and the above constructs were transfected into 293T cells. All experiments were repeated three times. *p < 0.05, **p < 0.01, ***p < 0.001. *Ctrl-ev:* EVs derived from PBS-treated ACs, *Cae-ev:* EVs derived from caerulein-treated ACs, *NC-mimic:* macrophages transfected with NC-mimic, *miR-183-5p mimic:* macrophages transfected with miR-183-5p mimic, *NC-inhibitor:* macrophages transfected with NC-inhibitor, *miR-183-5p inhibitor:* macrophages transfected with miR-183-5p inhibitor, *si-NC+miR-183-5p inhibitor:* macrophages transfected with miR-183-5p inhibitor and si-NC, *siFoxO1+miR-183-5p inhibitor:* macrophages transfected with miR-183-5p inhibitor and siFoxO1.

The downstream target genes of miR-183-5p were predicted using online analysis tools, including TargetScan (http://www.targetscan.org), miRWalk (http://mirwalk.umm.uni-heidelberg.de) and miRDB (http://www.mirdb.org). FoxO1 is one of the 118 genes predicted by all three tools, and there is evidence that it is closely related to macrophage polarization (**Figure 5A**). TargetScan revealed that the 3’UTR of FoxO1 contains a complementary binding site for miR-183-5p (**Figure 5B**). We exposed macrophages to EVs from ACs treated with PBS or caerulein to verify that EV miR-183-5p regulated macrophage polarization by targeting FoxO1 in macrophages. Western blot analysis showed that EVs derived from caerulein-treated ACs decreased the levels of FoxO1 and P-FoxO1 (**Figure 5C**). Subsequently, we confirmed the relationship between miR-183-5p and FoxO1 and P-FoxO1 levels by overexpressing or silencing miR-183-5p in NR8383 macrophages and BMDMs, respectively. Western blot analysis showed decreased FoxO1 and P-FoxO1 levels in cells overexpressing miR-183-5p, but their levels were significantly increased by the miR-183-5p inhibitor (**Figure 5D**). The results of immunofluorescence staining also confirmed that FoxO1 levels were decreased in BMDMs overexpressing miR-183-5p, but significantly increased in BMDMs with miR-183-5p knockdown (**Supplemental Figure 5A**). Moreover, the transfection of macrophages with the miR-183-5p inhibitor and siFoxO1 ameliorated the inhibitory effect of miR-183-5p and increased iNOS expression (**Figure 5E**). To confirm this hypothesis, we used dual-luciferase reporter gene assays. Compared with the 3’UTR-FoxO1-mutant group, the 3’UTR-FoxO1 group showed significantly reduced luciferase activity, indicating that miR-183-5p directly targets FoxO1 (**Figure 5F**). In summary, miR-183-5p can directly target FoxO1 and promote M1 macrophage polarization.

### AC-Derived EV miR-183-5p Aggravates Pancreatitis in AP Rats *in vivo*

**Figure 6:**
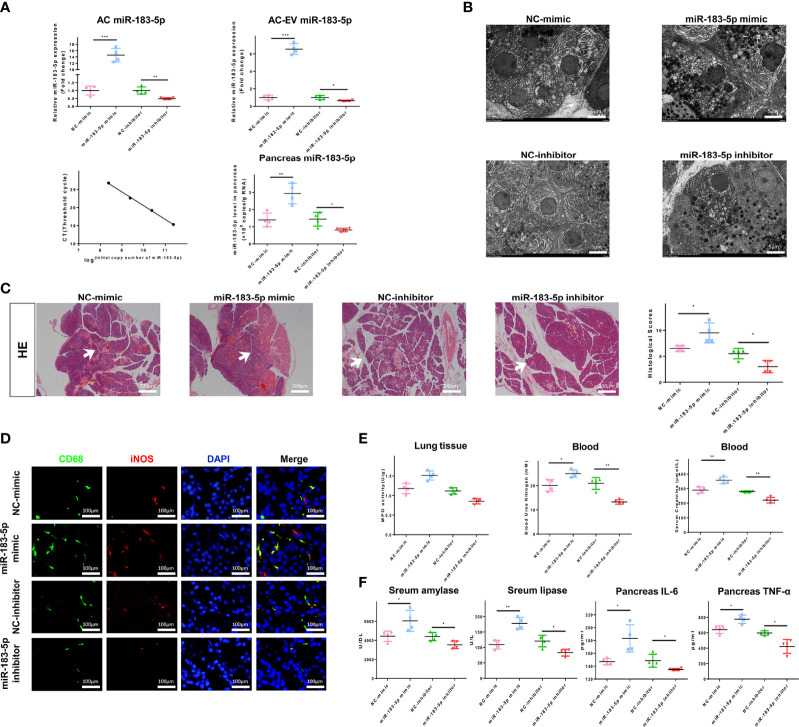
AC-derived EV miR-183-5p aggravates pancreatitis in AP rats *in vivo*. **(A)** Overexpression and inhibition of miR-183-5p expression in ACs and EVs were detected using qRT-PCR. The absolute expression of miR-183-5p in pancreatic tissue was calculated from a standard curve of logarithmic values of miR-183-5p concentrations and the CT value. **(B)** Electron microscopy photographs of pancreatic sections obtained from the above groups of AP rats. Scale bar, 10 μm. **(C)** Representative photographs and histological scores of HE-stained pancreatic sections obtained from AP rats in the following groups at 6 h after the EV injection s: NC-mimic, NC-inhibitor, miR-183-5p mimic and miR-183-5p inhibitor. Pancreatic injury is indicated by the white arrows. Scale bar, 200 μm. **(D)** Microscopy images of the distribution of CD68 and iNOS immunostaining in the indicated colors in pancreatic sections from the aforementioned groups of AP rats. Scale bars, 100 μm. The fold change in the number of macrophages co-expressing CD68 and iNOS in the four groups was determined. Scale bar, 400 μm. **(E)** Creatinine and urea nitrogen levels in the blood and MPO levels in the pancreas were determined. **(F)** Levels of amylase and lipase in the blood and inflammatory cytokines in the pancreas from the aforementioned groups of AP rats were determined by ELISA. All experiments were repeated three times. *p < 0.05, **p < 0.01, ***p < 0.001. *NC-mimic:* AP rats treated with EVs from acinar cells transfected with the NC-mimic, *miR-183-5p mimic:* AP rats treated with EVs from acinar cells transfected with the miR-183-5p mimic, *NC-inhibitor:* AP rats treated with EVs from acinar cells transfected with the NC-inhibitor, *miR-183-5p inhibitor:* AP rats treated with EVs from acinar cells transfected with the miR-183-5p inhibitor.

EVs were extracted from ACs transfected with miR-183-5p mimic or inhibitor and injected into AP rats, which were sacrificed 6 h later, to verify the function of AC-derived EV miR-183-5p *in vivo*. First, we examined the efficiency of the overexpression and inhibition of miR-183-5p in ACs, EVs and pancreatic tissue. Transfection of the miR-183-5p mimic significantly increased the level of miR-183-5p in ACs and AC-derived EVs, while transfection of the miR-183-5p inhibitor produced the opposite result. We injected EVs with miR-183-5p overexpression or knockdown into AP rats, which effectively increased or decreased the level of miR-183-5p in pancreatic tissue (**Figure 6A**). Electron microscopy results showed that EVs from ACs that overexpressed miR-183-5p exacerbated nuclear membrane contraction, endoplasmic reticulum swelling, and disordered arrangement (**Figure 6B**). Most importantly, EVs from miR-183-5p inhibitor-treated ACs alleviated AP and decreased the proportion of macrophages co-expressing CD68 and iNOS, while EVs that overexpressed miR-183-5p aggravated AP and increased the proportion of macrophages co-expressing CD68 and iNOS (**Figures 6C, D**). However, AC-derived EVs with miR-183-5p overexpression and silencing did not directly damage ACs (**Supplemental Figure 6A**), and thus EVs damaged pancreatic tissue by inducing M1 macrophage polarization. In addition, EVs from miR-183-5p mimic-treated ACs even damaged the lung and kidney (**Supplemental Figure 6B**). The levels of MPO in lung tissue and blood creatinine and urea nitrogen levels further confirmed that miR-183-5p-enriched EVs may aggravate lung and kidney damage (**Figure 6E**). Subsequently, we measured lipase and amylase levels in the blood and inflammatory cytokine levels in the pancreas, and the results showed that EVs overexpressing miR-183-5p increased the levels of lipase, amylase, IL-6 and TNF-α (**Figure 6F**). These results indicate that miR-183-5p is delivered to pancreatic tissue through AC-derived EVs and aggravates AP by inducing M1 macrophage polarization.

### Macrophages Overexpressing miR-183-5p Aggravate Acinar Cell Damage

**Figure 7:**
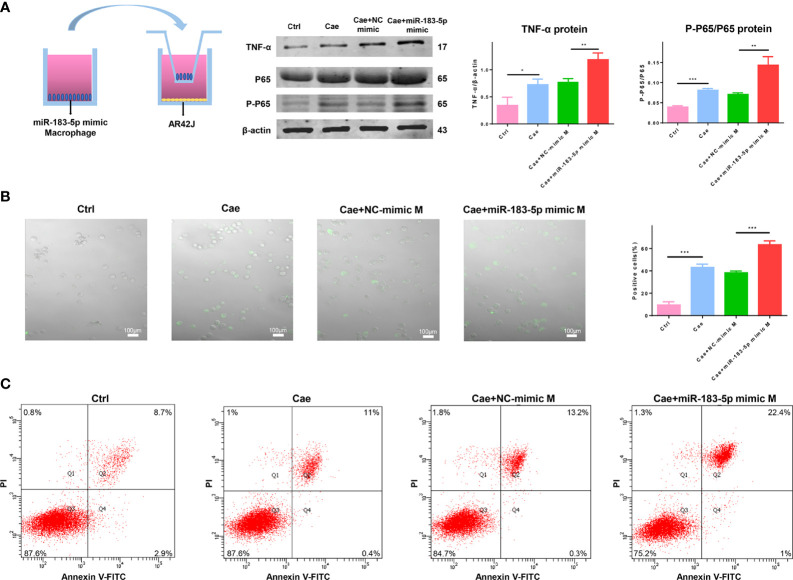
Macrophages overexpressing miR-183-5p aggravate AC damage. **(A)** Representative western blot and quantification of P-P65/P65 and TNF-α levels in ACs from the four groups: Ctrl, Cae, Cae+NC-mimic M, and Cae+miR-183-5p mimic M. **(B)** Trypsinogen activation assay in ACs from the above groups. The percentage of positive cells (white arrow) was calculated to quantify the degree of trypsinogen activation. **(C)** Representative flow cytometry results for necrosis in ACs from the above groups. All experiments were repeated three times. *p < 0.05, **p < 0.01, ***p < 0.001. *Ctrl:* PBS-treated acinar cells, *Cae:* caerulein-treated acinar cells, *Cae+NC-mimic: M* caerulein-treated acinar cells cocultured with macrophages transfected with the NC-mimic, *Cae+miR-183-5p mimic M:* caerulein-treated acinar cells cocultured with macrophages transfected with the miR-183-5p mimic.

We cocultured such macrophages with caerulein-treated ACs to prove whether macrophages overexpressing miR-183-5p damaged acinar cells. Cocultivation with miR-183-5p-overexpressing macrophages increased the ratio of P-P65/P65 and TNF-α levels in caerulein-treated ACs (**Figure 7A**). As a method to explore the degree of trypsinogen activation in acinar cells, we used the trypsin substrate BZiPAR to detect trypsin activation and the proportion of positive cells, which represented the degree of trypsin activation. The results showed that macrophages overexpressing miR-183-5p increased trypsinogen activation in caerulein-treated ACs (**Figure 7B**). Subsequently, flow cytometry was performed to further verify the damage to acinar cells, and the proportion of necrotic ACs was significantly increased after coculture with miR-183-5p-overexpressing macrophages (**Figure 7C**). Our results show that overexpressing miR-183-5p in macrophages induces trypsinogen activation and aggravates acinar cell damage.

### Increased EV miR-183-5p Expression is Detected in Serum EVs and Positively Correlates with AP Severity

**Figure 8:**
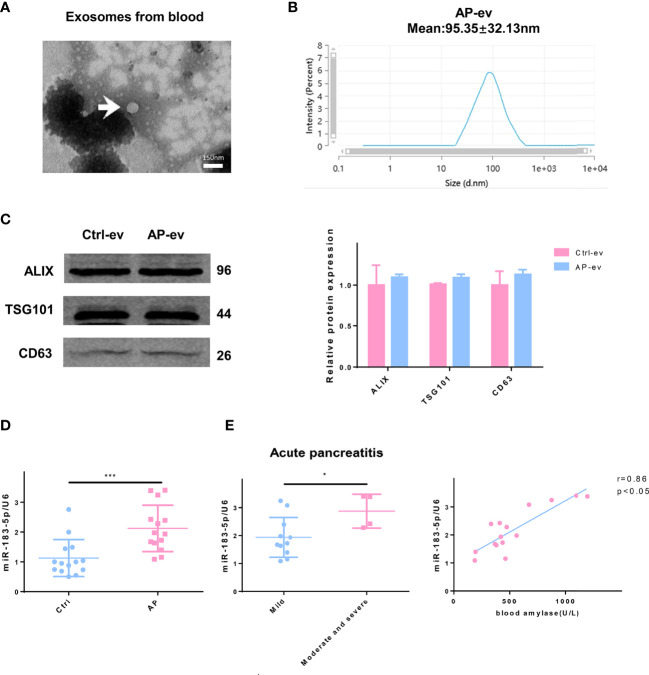
Higher EV miR-183-5p levels in blood from patients with AP positively correlate with disease severity. **(A)** Representative electron micrograph of EVs purified from blood collected from patients with AP. EVs are labeled with white arrows. Scale bar, 150 μm. **(B)** NTA of the diameter of EVs in the blood of patients with AP. **(C)** Western blot analysis of the protein expression of EV markers (including Alix, TSG101 and CD63) in EVs isolated from blood. **(D)** The level of miR-183-5p in EVs from blood of normal controls and patients with AP. **(E)** The level of miR-183-5p in EVs from blood of patients with mild and moderate-to-severe AP. Correlation analysis between miR-183-5p expression in blood EVs and blood amylase levels in patients with AP (r = 0.86, p < 0.05). All experiments were repeated three times. *p < 0.05, **p < 0.01, ***p < 0.001. *Ctrl-ev:* EVs from the serum of normal participants, *AP-ev:* EVs from the serum of patients with AP, *mild group:* patients with mild AP, *moderate-to-severe group:* patients with moderate or severe AP.

We isolated and purified EVs present in blood samples from patients with AP (n=15) and normal individuals (n=15) to confirm the clinical significance of our findings. EVs were discoid vesicles between 50 and 150 nm in diameter, as determined using TEM (**Figure 8A**). NTA showed that the mean particle size of EVs purified from serum was 95.35 ± 32.13 nm (**Figure 8B**). The EV protein markers Alix, TSG101 and CD63 were also used to identify EVs by performing western blotting, which confirmed that the extracted material was EVs (**Figure 8C**). qRT-PCR results showed a significant increase in miR-183-5p levels in serum EVs from patients with AP compared with those of normal individuals (**Figure 8D**). Lower levels of miR-183-5p were detected in the blood of patients with mild to moderate pancreatitis than in patients with severe pancreatitis. The blood amylase levels represent the disease severity, and the results showed that the EV miR-183-5p in blood was positively correlated with the amylase level (**Figure 8E**).

## Discussion

The structural and functional integrity of acinar cells is an important factor associated with the severity of AP. As shown in our previous studies, the regulation of necrosis, autophagy, apoptosis and energy stress of acinar cells is a promising approach in the management of AP (15–17). However, the mechanism of AP is still not well elucidated. In the present study, AC-derived EV miR-183-5p aggravated AP by promoting M1 macrophage polarization through downregulation of FoxO1, which provides a new perspective to understand AP.

Previous research has mainly focused on EVs in the circulating blood of patients with AP. These EVs contain a large number of miRNAs with proinflammatory functions, which damage other organs, in addition to the pancreas (18, 19). No reports have described the function of acinar cell-derived EVs in AP. Our study showed that EVs from caerulein-treated ACs increased M1 macrophage infiltration of the pancreatic tissue in AP rats and aggravated pancreatic tissue injury compared with control EVs. In general, EVs not only regulate inflammation in the pancreas itself but also contribute to the damage to other organs and thus play a vital immunoregulatory role in the progression of pancreatitis.

Macrophages have a high degree of heterogeneity, plasticity and diversity, and differences in the microenvironment and exogenous stimuli can induce polarization into two activation states (M1 and M2) with different phenotypes and biological functions (20). Wu et al. showed that M1 macrophages were dominant in the proinflammatory stage of AP, while M2 macrophages were dominant during pancreatic repair/regeneration (5). Either increasing the polarization of M1 macrophages or inhibiting the polarization of M2 macrophages may aggravate inflammation. In our study, in the inflammatory state, EVs aggravated pancreatitis mainly by increasing the number of M1 macrophages in pancreatic tissue, but did not affect M2 macrophages. DAMPs released by damaged acinar cells are an important stimulus for M1 macrophage polarization (21). In addition, noncoding RNAs, proteins and other components carried by EVs are considered to induce macrophage polarization (22, 23). Therefore, we performed high-throughput sequencing of miRNAs in AC-derived EVs. Our results showed that ACs induce M1 macrophage polarization in the pancreas by secreting miR-183-5p-rich EVs. This EV-mediated pathway not only represents a new mechanism by which acinar cells and macrophages communicate to promote AP but also fills a gap in our knowledge of the regulation of AP by acinar cell-derived EVs.

MiR-183-5p plays an important regulatory role in a variety of diseases. Rider et al. showed that increasing miR-183-5p levels may induce epithelial cell damage and cause allergens to leak, thereby inducing the production of inflammatory factors and aggravating asthma (24). Other studies have also shown that miR-183-5p is significantly upregulated in acute lung injury and localized aggressive periodontitis and plays an important role in the progression of inflammatory diseases (25, 26). FoxO1, one of the most well-known members of the FoxO family, has multiple functions, such as regulating the immune response, apoptosis, autophagy, oxidative stress, and the cell cycle (27–30). Liu et al. showed that in nonalcoholic fatty liver disease, the inhibition of FoxO1 in macrophages by miR-192 resulted in the release of a large number of proinflammatory cytokines, leading to M1 polarization and aggravating the progression of liver inflammation (31). Our research showed that EV miR-183-5p derived from ACs may induce M1 macrophage polarization by inhibiting FoxO1, thus aggravating pancreatitis. We propose that the EV miR-183-5p/FoxO1 axis is a new target for regulating the immune response in individuals with AP.

As an important part of the innate immune system, macrophages play roles in antigen presentation and the release of various inflammatory cytokines, which are important in the pathophysiological processes of inflammation and metabolism and are also key factors for physiological homeostasis (20, 32). Macrophages are mainly polarized to the M1 type, which plays an important role in facilitating the continuous development and amplification of the inflammatory response during the natural course of AP. Our experimental results also confirmed that macrophages overexpressing miR-183-5p increased the abnormal activation of zymogen in ACs and further aggravated AP.

The occurrence and development of excessive inflammatory responses in AP are dynamic inter-related processes involving multiple factors. Acinar cell injury and M1 macrophage polarization are the initiating and promoting factors, respectively. In the crosstalk between acinar cells and macrophages, damage to a single node often leads to continuous deterioration of the disease. The ideal management of AP should aim at breaking this positive feedback loop. Among the possibilities, inhibiting M1 macrophage polarization can effectively curb the amplification effect on local pancreatic inflammatory damage, thereby inhibiting the progression of an excessive inflammatory response in AP, and is thus a potentially effective therapeutic target for inflammatory damage.

Lipase and amylase are common serum parameters in the diagnosis of AP in daily practice. However, both lipase and amylase have a short diagnostic window and lack the ability to indicate the severity of AP. As shown in the present study, the EV miR-183-5p expression level in the blood was higher in patients with pancreatitis than in the normal group, and miR-183-5p levels in EVs were positively correlated with disease severity. EV miR-183-5p has a great potential as a biomarker that may represent an innovative direction in the future diagnosis of AP. In addition, EV miR-183-5p secreted by ACs not only causes M1 macrophage polarization in the pancreas, thereby aggravating pancreatitis, but may also travel through circulation to multiple organs or systems, such as the lungs and kidneys, providing a new research direction for exploring multiple organ failure in individuals with AP.

In conclusion, AC-derived EV miRNA-183-5p aggravates AP by promoting M1 macrophage polarization through downregulation of FoxO1, thus providing a new perspective in the understanding of AP and potentially an innovative target in the management of AP.

## Data Availability Statement

The datasets presented in this study can be found in online repositories. The name of the repository and accession number can be found below: NCBI Sequence Read Archive; PRJNA833775.

## Ethics Statement

The studies involving human participants were reviewed and approved by the Ethics Committee of the First Affiliated Hospital of Harbin Medical University. The patients/participants provided their written informed consent to participate in this study. The animal study was reviewed and approved by the Ethics Committee of the First Affiliated Hospital of Harbin Medical University.

## Author Contributions

D-sT and FC performed AP modeling and EV extraction, interpreted the results and wrote the manuscript. C-sY and J-tC performed TEM, IF, H&E staining and biochemical analyses. X-yG and LC performed cell culture and transfection. LL performed statistical analysis and the luciferase reporter assay. Y-lL and J-mM fed the rats and performed the necrosis assay, KF and LG performed western blotting and qRT-PCR. N-sR and BS performed flow cytometry. LJ provided conceptual advice and revised the manuscript. GW conceived the project and supervised the study. All authors contributed to the article and approved the submitted version.

## Funding

This study was supported by grants from the National Natural Science Foundation of China (Nos. 81770639, 82070657 and 81900586), Application Technology Research and Development Project of Heilongjiang Province (No. GA20C019), First Affiliated Hospital of Harbin Medical University Fund for Distinguished Young Scholars (Nos. HYD2020JQ0006 and HYD2020JQ0010), Research Project of Chinese Research Hospital Association (Y2019FH-DTCC-SB1), Innovative Research Funds of the First Affiliated Hospital of Harbin Medical University (2018BS013) and Postdoctoral Fund of Heilongjiang Province (LBH-Z18131).

## Conflict of Interest

The authors declare that the research was conducted in the absence of any commercial or financial relationships that could be construed as a potential conflict of interest.

## Publisher’s Note

All claims expressed in this article are solely those of the authors and do not necessarily represent those of their affiliated organizations, or those of the publisher, the editors and the reviewers. Any product that may be evaluated in this article, or claim that may be made by its manufacturer, is not guaranteed or endorsed by the publisher.
