# Supplementary material for: Acinar Cell-Derived Extracellular Vesicle MiRNA-183-5p Aggravates Acute Pancreatitis by Promoting M1 Macrophage Polarization Through Downregulation of FoxO1
Source: Front Immunol. 2022 Jul 13;13:869207. doi: 10.3389/fimmu.2022.869207 (PMC9326086; doi:10.3389/fimmu.2022.869207)
Supplement: Supplementary file 1 [file DataSheet_1.docx]

**Supplementary Data**

**6 Supplementary Figure**

**1 Supplementary Table**

| Supplemental Table 1 Primer sequences for RT-qPCR | | |
| --- | --- | --- |
|  | Forward | Reverse |
| Rat iNOS | TCAGCTACGCCTTCAACACCA | GGCCAA ATACCGCATACCTGA |
| Rat Arg-1 | CTGCATATCTGCCAAGGACATC | GTTCCCCAGGGTCCACATC |
| Rat Il-6 | GTTCTCTGGGAAATCGTGGA | TGTACTCCAGGTAGCTATGG |
| Rat Il-1β | CAGGATGAGGACATGAGCACC | CTCTGCAGACTCAAACTCCAC |
| Rat TNF-α | TCTCATCAGTTCTATGGCCC | GGGAGTAGACAAGGTACAAC |


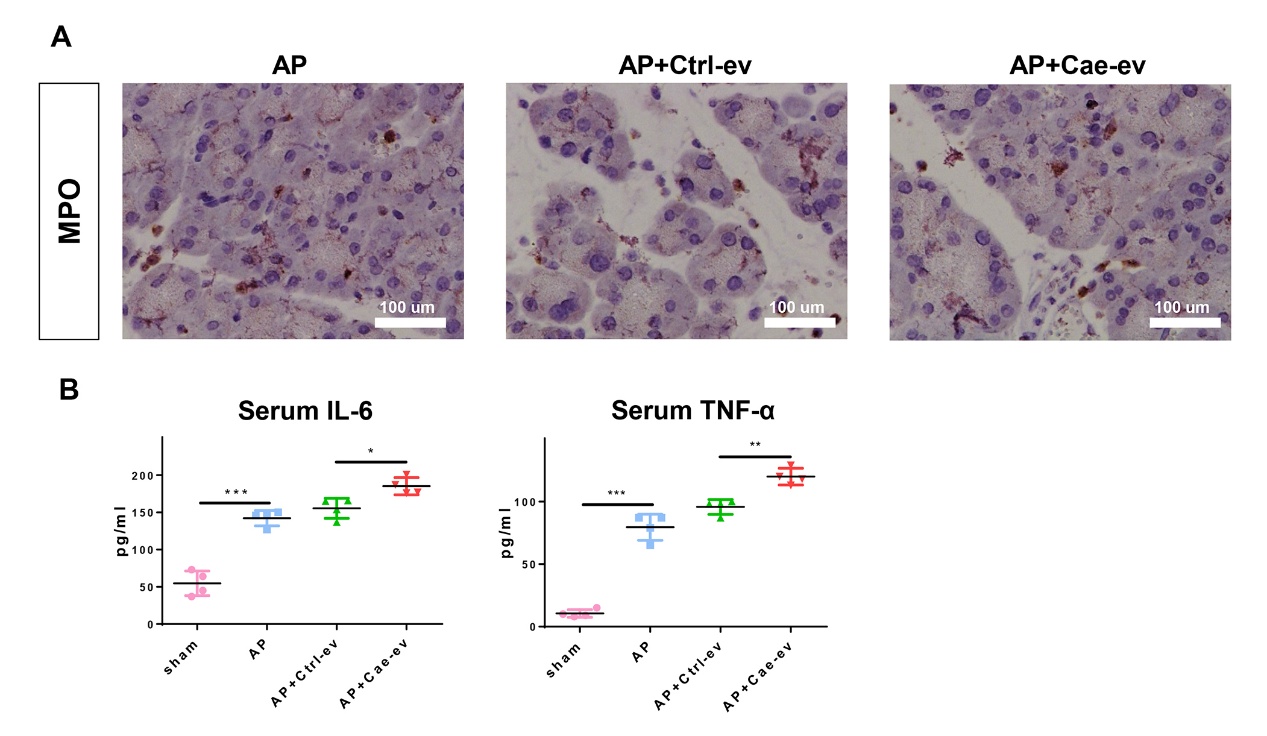


**Supplemental Figure 1: Effects of extracellular vesicles on intrapancreatic neutrophils and inflammatory factors in circulating blood of AP rats.**

(A) Representative photographs MPO stained pancreatic sections harvested from the rats in the 3 groups 6 h after EV injection: AP, AP+Ctrl-ev and AP+Cae-ev. Scale bar, 100 μm.(B) Inflammatory cytokine levels in peripheral blood were determined by ELISA. Data are presented as the mean ± SD. All experiments were repeated three times. **p* < 0.05, ***p* < 0.01. *ACs* acinar cells, *AP* acute pancreatitis, *ev* EVs, *Ctrl-ev* EVs from acinar cells, *Cae-ev* EVs from caerulein-treated acinar cells.


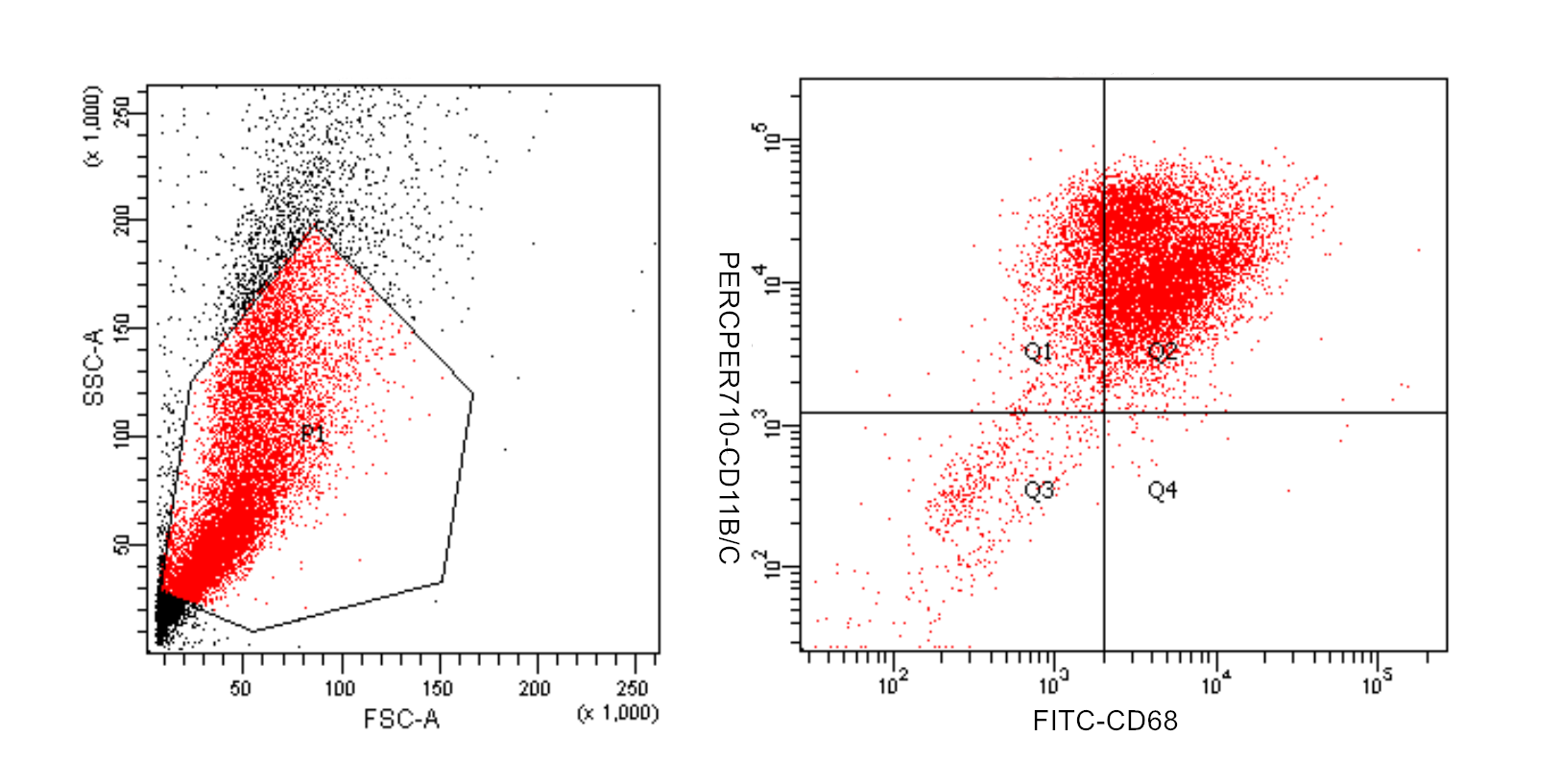


78.8%

16.1%

4.8%

0.3%

**Supplemental Figure 2: Identification of macrophages.** Monocyte from the rat bone marrow and transformed them into macrophages by treatment with M-CSF. Flow cytometry analysis to quantify the number of CD68 and CD11b/c cells (macrophage markers).

**Supplemental Figure 3: Overexpression of miRNAs in macrophages.**

The overexpression of miR-144-3p, miR-135b-5p, miR-126a-5p, miR-183-5p and miR-451-5p in macrophages transfected with miRNA mimic was detected by qRT-PCR. Data are presented as the mean ± SD. All experiments were repeated three times.


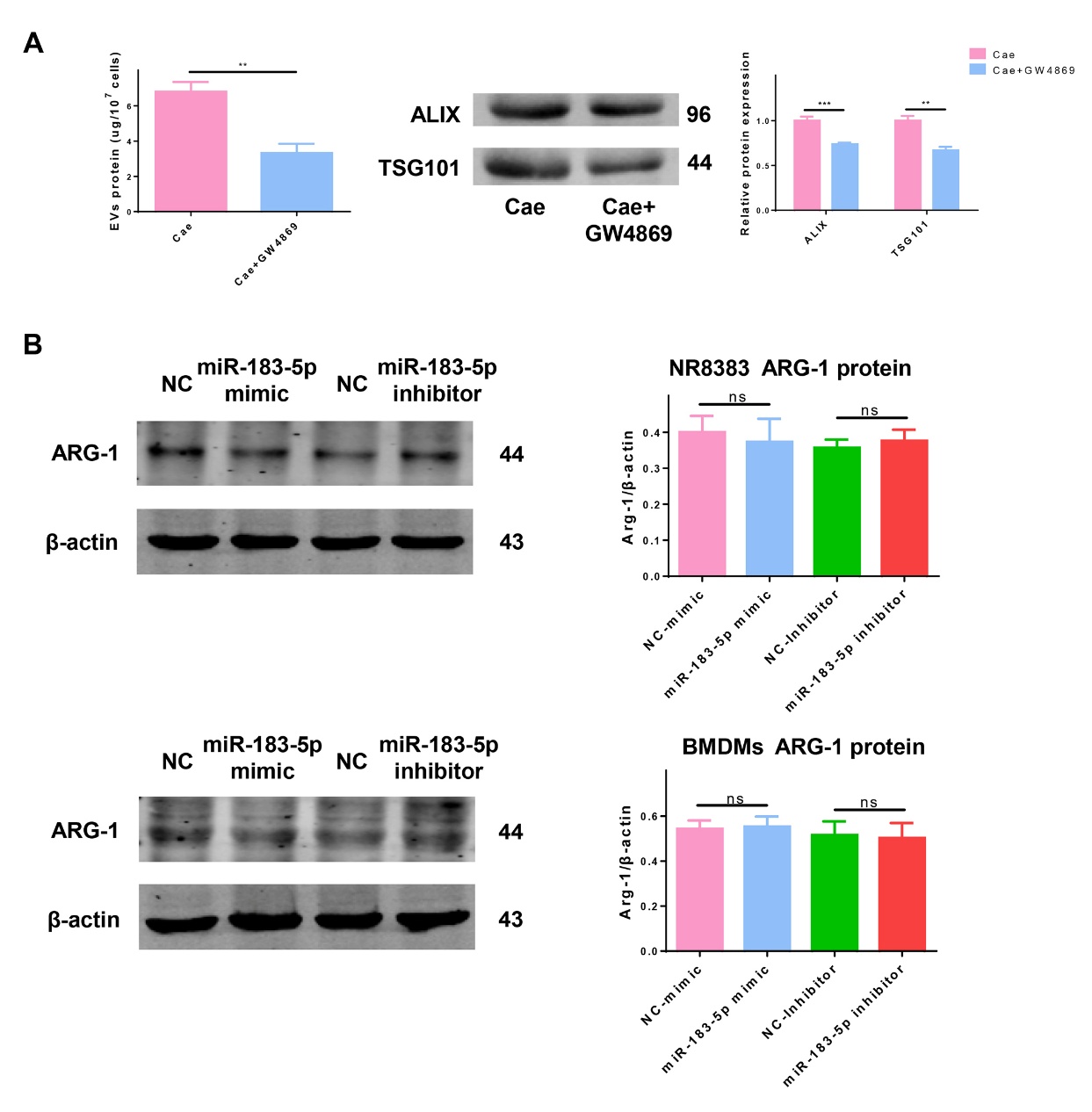


**Supplemental Figure 4: Effects of AC-derived EV miR-183-5p on M2 macrophage polarization.** (A)The concentrations of proteins in the Cae and Cae and GW4869 groups and the levels of Tsg101 and Alix in EVs were determined by western blotting. (B) Protein expression and analysis of Arg-1 in the above groups of NR8383 macrophages and BMDMs by western blotting. Data are presented as the mean ± SD. All experiments were repeated three times. **p* < 0.05, ***p* < 0.01, ****p* < 0.001. *Cae* caerulein-treated acinar cells, *Cae +GW4869* caerulein and GW4869 treated acinar cells, *NC-mimic* macrophages transfected with NC-mimic, *miR-183-5p mimic* macrophages transfected with miR-183-5p mimic, *NC-inhibitor* macrophages transfected with NC-inhibitor, *miR-183-5p inhibitor* macrophages transfected with miR-183-5p inhibitor, *BMDMs* bone marrow-derived macrophages.

**
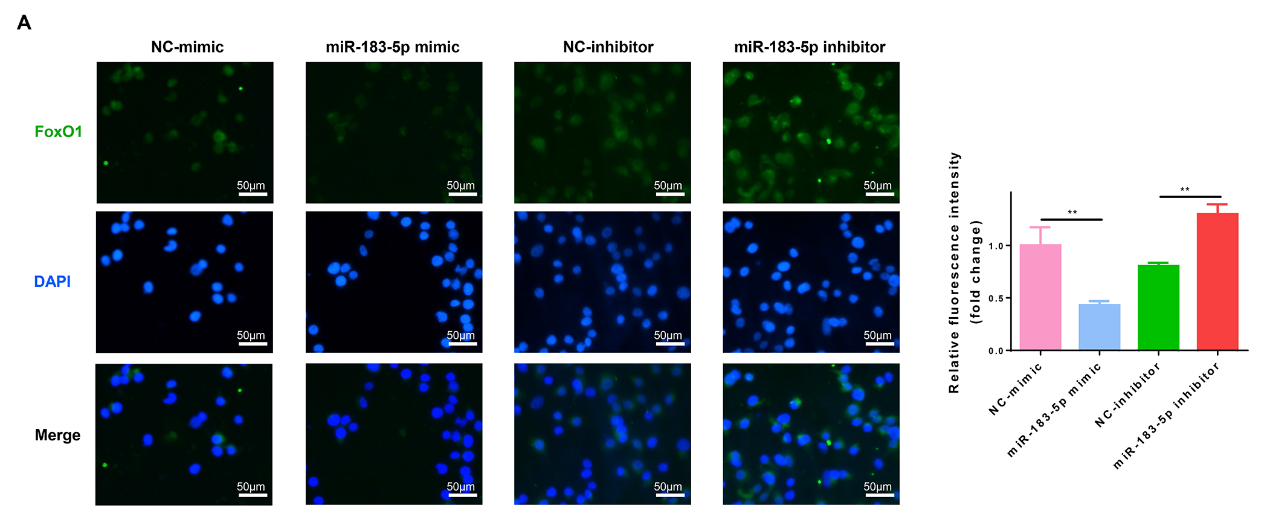
**

**Supplemental Figure 5:** **Macrophages overexpressing miR-183-5p inhibit FoxO1 expression.**

(**A**) Microscopy of the distribution of FoxO1 immunostaining in the indicated colors in BMDMs. The changes of FoxO1 in four groups of BMDMs were determined. Scale bar, 50 μm. ***p* < 0.01. *NC-mimic* BMDMs transfected with NC-mimic, *miR-183-5p mimic* BMDMs transfected with miR-183-5p mimic, *NC-inhibitor* BMDMs transfected with NC-inhibitor, *miR-183-5p inhibitor* BMDMs transfected with miR-183-5p inhibitor.


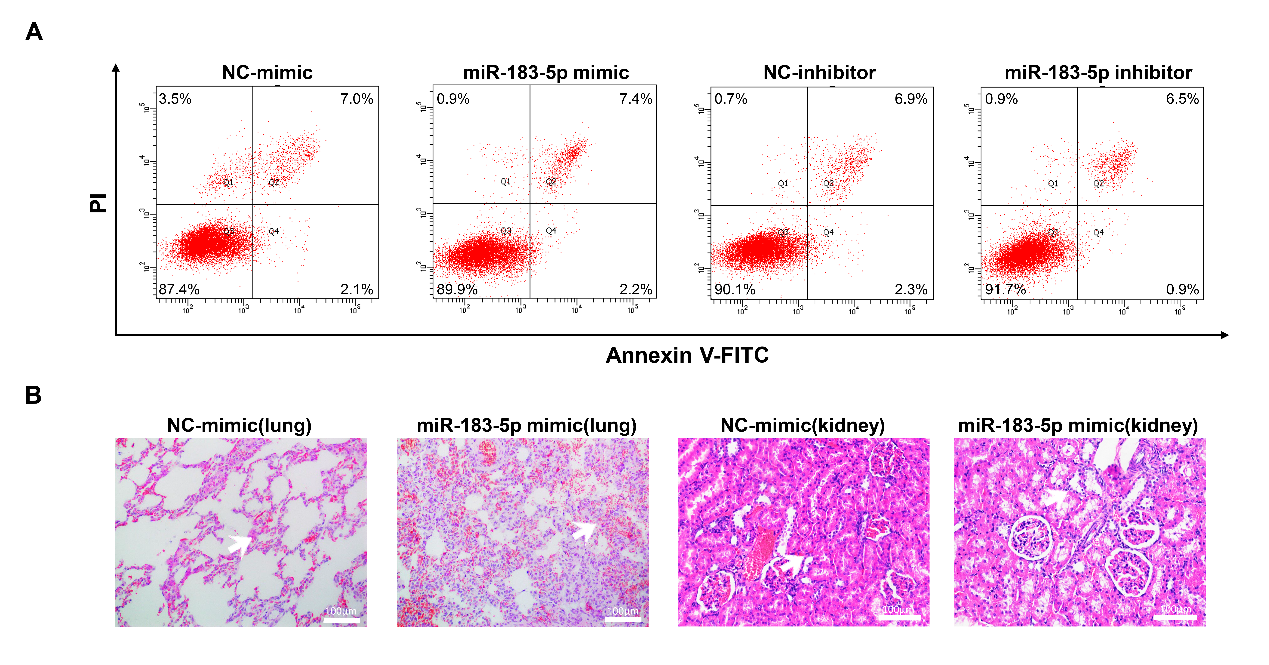
**Supplemental Figure 6: Effects of extracellular vesicles derived from acinar cells overexpressing miR-183-5p on acinar cells, lung and kidney.**

(**A**) Representative flow cytometry results for necrosis in ACs treated with AC-derived Evs from the four groups: NC-mimic, miR-183-5p mimic, NC-inhibitor and miR-183-5p inhibitor. (**B**) Representative photographs and histological scores of HE-stained lung and kidney sections obtained from AP rats 6 h after EV injection in the following two groups: NC-mimic and miR-183-5p mimic. Tissue injury is indicated by the white arrows. Scale bar, 100 μm. All experiments were repeated three times. **p* < 0.05, ***p* < 0.01, ****p* < 0.001. *NC-mimic* acinar cells treated with EVs from acinar cells transfected with the NC-mimic, *miR-183-5p mimic* acinar cells treated with EVs from acinar cells transfected with the miR-183-5p mimic, *NC-inhibitor* acinar cells treated with EVs from acinar cells transfected with the NC-inhibitor, *miR-183-5p inhibitor* acinar cells treated with EVs from acinar cells transfected with the miR-183-5p inhibitor.
